# Evaluation of antioxidant activity of three common potato (*Solanum tuberosum)* cultivars in Iran

**Published:** 2012

**Authors:** Faride Hesam, Gholam Reza Balali, Reza Taheri Tehrani

**Affiliations:** 1 *Faculty of Food Science, Islamic Azad University, Shahreza Branch, Isfahan, Shahreza, I. R. Iran *; 2 *Department of Potato Research Biotechnology, University of Isfahan, I. R. Iran*

**Keywords:** Antioxidant activity, Potato, Radical scavenging, Total phenolic compounds

## Abstract

**Objectives: **Potato *(Solanum* *tuberosum *L.), as a whole food, contains high levels of vitamins and important antioxidants including phenolic acids, carotenoids and flavonoids. The objective of this study was to determine the total phenolic content and antioxidant activities of three common potatoes (*Solanum tuberosum*) cultivars in Iran i.e., Savalan, Agria and Sante.

**Materials and Methods: **Phenolic compound extraction of samples was done with methanol and total phenolic on the basis of folin-ciocalteu assay was estimated as 16.58 to 36.24 mg GAE/100g dry sample. The antioxidant activities of potato extracts on the basis of inhibition of linoleic acid peroxidation and DPPH assay were compared with a commercially available antioxidant, α -tocopherol.

**Results: **Savalan had the highest phenolic content and the highest DPPH radical scavenging activity with EC_50_ value of 41.815±mg/ml (DB). Also Savalan had the best inhibitory action against linoleic acid oxidation at 94.10±1.89% at 50 mg/ml sample concentration. Methanolic potato extracts had better antioxidant activity than α-tocopherol. Significant (p<0.01) negative correlation was observed between total phenolic content and the EC_50_ for DPPH radical scavenging activity(R=-0.877), but there was no correlation between total phenolic content and total antioxidant activity.

**Conclusion: **Metanolic extracts of three potato cultivars are able to inhibit the oxidation process. The correlation between total phenolic content and DPPH radical scavenging activity indicates that phenolic compounds are responsible for antiradical activity.

## Material and Methods

**Sample (Preparation of sweet potato flour)**

Three potato cultivars ‘Savalan, Sante and Agria’ were obtained from Potato Research Biotechnology Department, university of Isfahan. The tubers were washed and cut into 2-cm slices. After steaming for 15 min at 100°C (to prevent browning of the flesh), the slices were allowed to cool at room temperature. The flesh was then removed from the peel by hand, freeze-dried (DW8, Heto Holten, Denmark) and ground to fine powder. The prepared flour was kept at 4° C until use.

**Extraction of phenolic compounds**

Phenolic compound extraction was carried out according to the method of Romboea et al. (2009). A volume of 80 ml methanol was added to ten grams of potato flour to produce a slurry suspension and kept overnight in room temperature. The prepared suspension was filtered using Whatman No.1 filter paper and the filtrate was diluted to 100 ml by adding methanol. Sample solutions were stored at 4 °C in amber bottles and used as the stock solution (100 mg/ml) for subsequent analyses.

**Determination of total phenolic compounds using Folin-Ciocalteu Phenolic reagent**

The total phenolic compounds in the potato extracts were determined with Folin-Ciocalteu reagent according to the method of Slinkard & Singleton (1997) ▶, using gallic acid as a standard phenolic compound. A volume of 200 microlitres of the sample was mixed with 1.4 ml distilled water and 100 µl of Folin–Ciocalteu reagent. After at least 30 seconds but not more than 8 minutes, 300 µl of 20% Na2CO3 solution was added and the mixture was allowed to stand for 2 h. The absorbance was measured at 765 nm with Biowaveп UV–Vis Spectrophotometer (Biochrom WPA, England). Standard solutions of Gallic acid (10 – 100 ppm) were similarly treated to prepare the calibration curve. The amount of total phenolic compounds in the potato extracts was determined in mg Gallic acid equivalent (GAE) /100 g dry sample, using the equation obtained from the standard Gallic acid graph. For each cultivar, five replications were considered. 

**Antioxidant activity by ferric thiocyanate method**

The antioxidant activity was determined according to the ferric thiocyanate method in linoleic acid emulsion (Huang et al., 2006 ▶). One ml aliquot of a 50 mg/ml sample solution was mixed with 2 ml of 0.05 M phosphate buffer pH 7.0, 1 ml of 2.51% (v/v) linoleic acid solution in 99.5% (v/v) ethanol, and 1 ml distilled water. The mixed solution was incubated in the dark at 40 °C. Every 24h during incubation period, 0.1 ml aliquot of the mixture was diluted with 9.7 ml of 75% (v/v) ethanol, followed by the addition of 0.1 ml of 30% (w/v) NH_4_SCN and 0.1 ml of 20 mM FeCl_2 _in 3.5% (v/v) HCl. After 3 min, the peroxide level was determined by reading the absorbance at 500 nm in a Biowaveп UV–Vis Spectrophotometer (Biochrom WPA, England). A control solution was prepared by substituting 1 ml methanol for the sample. These steps were repeated every 24 hours until the control reached its maximum absorbance value. Percent inhibition was calculated as follows:

1) % Activity= (1-(∆A_sample/_ ∆A_blank_)) × 100 

Where ∆A is the absorbance increase.

**Free radical scavenging activity measured by 1, 1-Diphenyl-2-picryl-hydrazil**

The scavenging activity of sweet potato extracts was measured by 1, 1-diphenyl- 2-picrylhydrazyl (DPPH) radical using the method of Huang et al. (2005b) ▶. Sample solutions with concentrations 10, 20, 30, 40 and 50 mg/mL were prepared from the stock solution. One ml of freshly prepared methanolic solution (80 ppm) of DPPH (1, 1-diphenyl-2-picryl-hydrazyl) was added to a 1 ml aliquot of samples. The mixture was kept in the dark for 30 minutes. Then, the absorbance was measured at 517 nm using Biowaveп UV–Vis Spectrophotometer (Biochrom WPA, England). The radical scavenging activity of alpha-tocopherol (10–50 ppm) was also determined. The capability to scavenge the DPPH radical was calculated using the following equation:

2) % Activity = (1-(A_sample/_ A_blank_)) × 100 

The EC_50_ value, which is the sample concentration at 50% activity, was determined by interpolation. 

**Statistical analysis**

Data were analyzed using univariate analysis of variance and Duncan’s multiple range test for post-hoc analyses. Statistical calculations were performed by SAS Statistical Program, (SAS Institute, 1999). p<0.05 was considered statistically significant. Pearson correlation (p<0.05) tests was used to determine the relationship between total phenolic content and the EC_50_ values for DPPH radical scavenging activity and antioxidant activity.

## Results

**Total phenolic content**

The amount of phenolic compounds of three cultivars of potato (Agria, Savalan and Sante) in Iran is listed in table 1 (the data are expressed as mg GAE/100 g sample). Phenolic content ranged from 16.57–36.24 mg GAE/100 g dry sample. There was significant difference among the methanolic extracts of potato cultivars (p< 0.05) in terms of phenolic content. Among the potato varieties, Savalan had the highest phenolic content followed by Sante and Agria (Table 1). 

**Table 1:** Total phenolic content of potato samples

**Cultivar**	**Total phenolic content (mg gallic acid equivalent/100 g dry sample)** d
Savalan	36.24±5.87^a^
Agria	16.57±8.56 c
Sante	30.36 ±4.93^b^

a–c These were statistically different (p<0.05)

d Each value is expressed as the mean±standard deviation (n = 5).

**Inhibition of linoleic acid oxidation**

The ferric thiocyanate method measures the amount of peroxide produced during the initial stages of oxidation which is the primary product of oxidation (Gülçin et al., 2007 ▶). Peroxide reacts with ferrous ion and produces ferric ion. The absorbance of a formed red-colored complex (ferric thiocyanate) is measured every 24 h until the reaction is complete (Elmastas et al., 2006 ▶). Inhibition by the methanolic potato extracts at 50 mg dry sample is shown in (Table 2). Savalan had the highest antioxidant activity. However, the difference between the antioxidant activities of the three potato varieties was statistically insignificant (p>0.05). In the present study, methanolic potato extracts had better inhibitory action than alpha-tocopherol on a µg analyte basis, the latter having an activity of 85.17% at 100 µg.

No significant correlation (r=-0.306, p>0.05) was found between total phenolic content and antioxidant activity of the potato extracts.

**Table 2:** Antioxidant activity of potato samples

**Cultivar**	**% Inhibition at 50 mg/mL**	**EC50 value (mg/mL sample, dry asis)** d
Savalan	94.10±1.89^a^	41.815-c
Agria	92.89±1.23 a	58.195^a^
Sante	91.36 ±4.93^a^	47.76^b^

a-c These were not statistically different (p>0.05)

d Each value is expressed as the mean±standard deviation (n = 5).

**DPPH radical scavenging activity**

**Figure 1:**
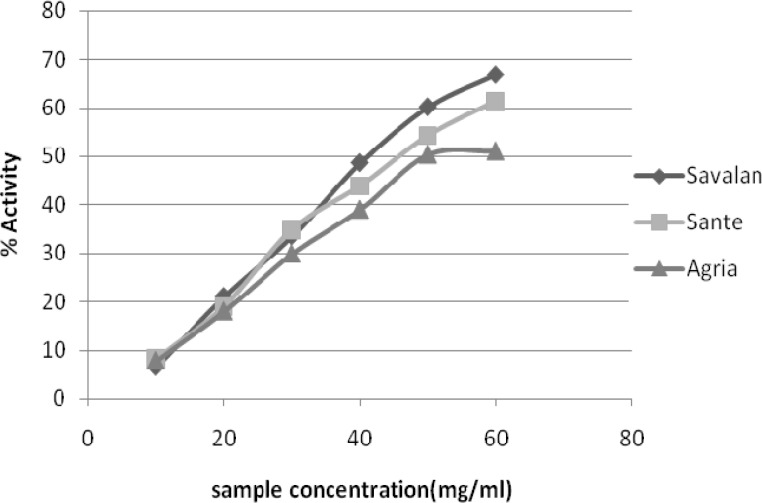
DPPH radical scavenging activity of methanolic potato extracts. Values are means of five trials.

The stable free radical DPPH has been widely used to test the free radical-scavenging ability of various dietary antioxidants (Brand-Williams et al., 1995 ▶). The reduction capability of the DPPH radical is determined by the decrease in its absorbance at 517 nm, induced by antioxidants (Elmastas et al., 2006 ▶; Singh & Rajini, 2004 ▶). Potato extracts exhibited marked DPPH free radical scavenging activity in a concentration-dependent manner (Figure 1). Antioxidant activity was evaluated with EC_50_ values, the concentration at which radical scavenging activity is 50%. The range of EC_50 _values of the analyzed potato varieties was 41.815– 58.195 mg/ml dry sample. Savalan had the highest scavenging activity, while Agria had the lowest. The EC_50_ for three potato extracts were significantly different (p<0.05) in terms of radical scavenging activity.

The radical scavenging activity of two potato samples (Savalan & Sante), on mg phenolic content basis, was higher than that of α tocopherol. A significant negative correlation (r=-0.856, p<0.01) for phenolic content and EC_50 _values was observed in potato varieties.

## Discussion

Phenolic content ranged from 16.57–36.24 mg GAE/100 g dry sample. Significant difference among varieties may be attributed to genotypes and harvest location which influence the accumulation of phenolic compounds by synthesizing different quantities and/or types of phenolics (Shahidi and Naczk, 1995 ▶; Romboao et al., 2009). 

Al-Saikhan et al. (1995) ▶ reported that potato contains 11.41-27.47 mg GAE/100g, while, Karadeniz et al. (2005) ▶ reported a phenolic content of 553±102.5 mg catechin/kg fresh weight (32.44-6.07 mg GAE/100 g). The amount of phenolic content in Philippians potatoes ranged 34-55 mg GAE/100 g dry matter (Rumbaoa et al., 2009). Higher values for phenolic content of potato were obtained by Kaur & Kapoor (2002) ▶ and Vinson et al. (1998), 231.46-9.73 mg GAE/100 g and 100.37-66.35 mg GAE/100 g, respectively. Some important factors such as sample treatment and extraction condition will affect the phenolic content of potato. Using of vigorous extraction methods, including homogenization, heating and hydrolysis, leads to higher yield according to literatures (Huang et al., 2005b ▶, Vinson et al., 1998; Kaur & Kapoor, 2002 ▶). Mohagheghi samarin et al. (2008) ▶ found that ultrasound can increase the yield of phenolic content extraction in potato peel. Vinson et al. (1998) estimated the amount of conjugated phenolics in potato at 57.9±13.4%. Higher yield of total phenolic content in the extract of Agria cultivar by Hamouz et al. (2007) ▶ relative to our study may be because of using ultrasound and vigorous shaking over the extraction period and also the condition and locality that their plant has been grown. However the data obtained from this study for total phenolic content of Agria cultivar is comparable to that of Reddivari et al. (2007) ▶.

The amount of antioxidant activity of methanolic extracts of potatoes in our study was between 92.89±1.23% and 94.10±1.89%. Rumbaoa et al. (2008) ranged antioxidant activity of Philippine potato varieties from 93.5±1.7 to 95.4±2.2% (by the same method of our article). Al-Saikhan et al. (1995) ▶ obtained antioxidant activity between 65.2% and 89.2% at 30 mg sample for yellow and white-fleshed varieties of potato. Activity of ethanolic and aqueous potato extract at 40 mg is 62.3% and 62.5%, respectively (Kaur & Kapoor, 2002 ▶).

Antioxidant activity of potato peel (Raja cultivar) was 31.60-61.91 % ( Mohagheghi samarin et al., 2007 ▶ ), which was evaluated on the basis of determination of the oxidative activity of potato peel extracts in refined soybean oil by the Rancimat method and oxidation was periodically assessed by measuring the peroxide and thiobarbituric acid. Karadeniz et al. (2005) ▶, on the other hand, reported an activity of 14.2-2.3% at 8 mg sample. Antioxidant activity values also depend strongly on the preparation of sample (leaching, extended steaming, lyophilisation) and the method used (ferric thiocyanate method) (Sulc et al., 2008 ▶). Insignificant correlation between total phenolic content and antioxidant activity of the potato extracts shows the presence of other non-phenolic constituents with antioxidative activity such as ascorbic acid in some varieties of potato (Al-Saikhan et al., 1995 ▶; Kalt, 2005 ▶).

The EC_50_ for three potato extracts were significantly different. Genotype and growth conditions, such as water availability, light quality and temperature, affect the synthesis and accumulation of phenolic compounds in some parts of the plant, and consequently, antioxidant activity (Reyes, 2005 ▶; Kalt, 2005 ▶). A significant negative correlation for phenolic content and EC_50_ values was observed in potato varieties, indicating that these phenolic compounds may contribute directly to the radical scavenging activity. This is consistent with the results of a similar study by Nara et al. (2006) ▶ and Reyes (2005) ▶ that reported correlation between phenolic content and antioxidant activity against free radicals. 

From our study it could be concluded that, metanolic extracts of three potato cultivars are able to inhibit the oxidation process. The correlation between total phenolic content and DPPH radical scavenging activity indicates that phenolic compounds are responsible for antiradical activity. Evaluating the antioxidant activity of other potato cultivars in Iran is recommended.
